# Fabrication and Characterization of Highly Sensitive Acetone Chemical Sensor Based on ZnO Nanoballs

**DOI:** 10.3390/ma10070799

**Published:** 2017-07-14

**Authors:** Qu Zhou, ChangXiang Hong, Yao Yao, Ahmed Mohamed Ibrahim, Lingna Xu, Rajesh Kumar, Sumaia Mohamed Talballa, S. H. Kim, Ahmad Umar

**Affiliations:** 1 College of Engineering and Technology, Southwest University, Chongqing 400715, China; hcx111000@163.com (C.H.); lingnaxu@cqu.edu.cn (L.X.); 2 College of Communication Engineering, Chengdu University of Information Technology, Chengdu 610225, China; yaoyao386@yahoo.com; 3 Department of Pharmaceutical Chemistry, Faculty of Pharmacy, Najran University, Najran 11001, Saudi Arabia; shakiroon4health@gmail.com; 4 Department of Chemistry, Jagdish Chandra DAV College, Dasuya 144205, Punjab, India; rk.ash2k7@gmail.com; 5 Department of Pathology, Faculty of Medicine, Najran University, Najran 11001, Saudi Arabia; omsuhieb@yahoo.com; 6 Department of Chemistry, College of Science and Arts, Najran University, P.O. Box 1988, Najran 11001, Saudi Arabia; semikim77@gmail.com; 7 Promising Centre for Sensors and Electronic Devices (PCSED), Najran University, P.O. Box 1988, Najran 11001, Saudi Arabia

**Keywords:** ZnO, nanoballs, acetone, current–voltage, electrochemical, sensor

## Abstract

Highly sensitive acetone chemical sensor was fabricated using ZnO nanoballs modified silver electrode. A low temperature, facile, template-free hydrothermal technique was adopted to synthesize the ZnO nanoballs with an average diameter of 80 ± 10 nm. The XRD and UV-Vis. studies confirmed the excellent crystallinity and optical properties of the synthesized ZnO nanoballs. The electrochemical sensing performance of the ZnO nanoballs modified AgE towards the detection of acetone was executed by simple current–voltage (*I*–*V*) characteristics. The sensitivity value of ∼472.33 μA·mM^−1^·cm^−2^ and linear dynamic range (LDR) of 0.5 mM–3.0 mM with a correlation coefficient (*R*^2^) of 0.97064 were obtained from the calibration graph. Experimental limit of detection (LOD) for ZnO nanoballs modified AgE was found to be 0.5 mM.

## 1. Introduction

ZnO nanomaterials has received exceptional attention and interest worldwide among the research fraternity due to their unique properties such as large surface to volume ratio, non-toxicity, ease of synthesis, *n*-type semiconducting nature, wide band gap of ~3.30 eV, large exciton binding energy, high thermal stability, excellent electrical, magnetic, catalytic properties, etc. [1,2,3,4,5]. A large variety of methods for synthesis of ZnO nanomaterials is reported in the literature which results in the formations of different morphologies like nano-mushrooms, fluffy nanoballs, nanorods, nanoribbons, nanowires, nanoflakes, nano/microspheres, nanocones, nanopillars, nano/micro flowers, nanoneedles, nanosheets, nanoaggregates, etc.

Among the various potential applications, real-time and reliable electrochemical sensing of harmful, toxic and explosive chemicals using ZnO nanostructured based electrochemical sensing, is widely studied. Such sensors offer advantages such as ambient stability, resistivity towards toxic and hazardous chemicals, chemical inertness, electrocatalytic activity and ease of fabrication. It has been reported that *n*-type semiconducting metal oxide nanomaterials enhance the rate of electron transfer between electrode and analyte molecules, which drastically improves the current response for target molecules [6]. Additionally, inorganic metal oxide nanoparticles serve as supra-molecular assembling units which provide large surface area for electrochemical sensing interface [7,8]. Electrical signals resulted from the interaction of the target analyte molecules and the ZnO nanostructured transducer layers, coated on the surface of the modified electrode, provide the valuable analytical information [9].

Toxic and highly hazardous chemicals such as nitrophenols [10,11], ammonia [12], CO [13], hydrazines [14,15], nitroanilines [16,17,18], hydrogen sulfide [19], ethanolamine [20], picric acid [21], ethyl acetate [22], ethanol [23], synthetic antioxidants and dyes in food articles [24,25], some bio-molecules like glucose [26,27,28], uric acid [29,30], urea [31,32], aspartic acid [33], dopamine [34], pH sensors [35], etc. have been detected and analyzed through electrochemical sensing techniques using ZnO modified electrochemical sensors. Recently, Ahmad et al. [18] reported a binder-free, stable, and highly efficient hydrazine chemical sensor based on vertically aligned ZnO nanorods directly grown on the surface of Ag electrode through a low-temperature solution process. The average diameter and length of ZnO nanorods were ~50 nm and 2.2 µm with a high aspect ratio of about 44. Excellent sensitivity of 105.5 µA·µM^−1^·cm^−2^ with a linear dynamic range of 0.01–98.6 µM and low detection limit of 0.005 µM. was observed. Unique lotus-leaf-like ZnO nanostructures deposited on FTO substrate showed very low-level detection of ethyl acetate with high sensitivity of ∼139.8 µA·mM^−1^·cm^−2^ and limit of detection of ∼0.26 mM [22]. Ameen et al. [36] synthesized ZnO nanowhiskers through a hydrothermal method and utilized them as electron mediators for the fabrication of electrochemical sensors for detecting p-hydroquinone. As fabricated p-hydroquinone chemical sensor exhibited a substantially high sensitivity of ~99.2 µA·µM^−1^·cm^−2^ with a very low detection limit of ~4.5 µM and linear dynamic range of ~10–200 µM. Ibrahim et al. [21] observed a high sensitivity of 24.14 μA·mM^−1^·cm^−2^ with good LDR of 0.078–10.0 mM against picric acid using electrochemical sensor based on ZnO nanostructures with cauliflower shaped morphologies. Tailoring the ZnO morphologies for acquiring large surface to volume ratio for better adsorption of the analyte species and hence fast charge transfer during the electrochemical process is one of the most critical and desired aspects of electrochemical sensing applications.

In the present work, a simple, low cost, and template-free hydrothermal method was adopted for the synthesis of ZnO nanoballs with highly rough surfaces. Morphological, structural, optical, crystal phases, vibrational and scattering properties of the ZnO nanoballs were evaluated through different analytic techniques. ZnO nanoballs were further utilized for the fabrication of highly sensitive acetone electrochemical sensors through *I*–*V* techniques. The ZnO nanoballs modified AgE showed the high sensitivity towards acetone.

## 2. Results and Discussion

### 2.1. Morphological, Structural, Optical and Compositions Properties of ZnO Nanoballs

**Figure 1:**
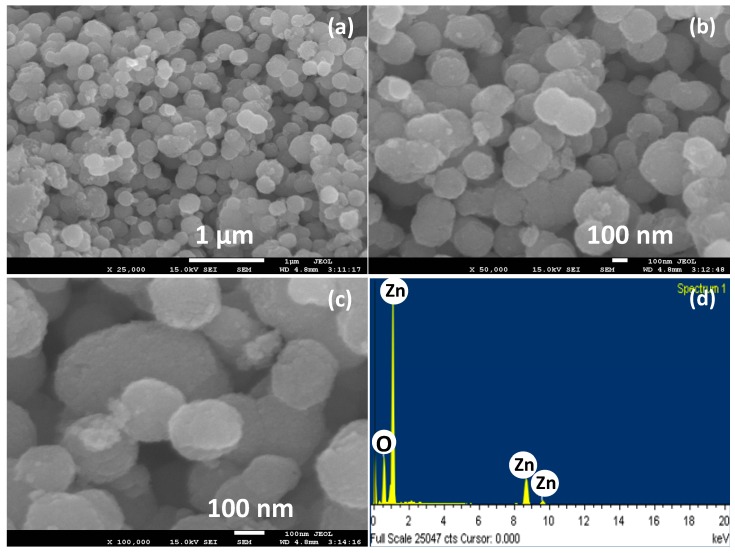
(**a**) Low magnification; and (**b**,**c**) high magnification FESEM images; and (**d**) EDS spectrum of ZnO nanoballs.

Figure 1 represents the field emission scanning electron microscopic (FESEM) images of the hydrothermally synthesized ZnO powders. Interestingly, almost ball shaped morphologies can be assigned to maximum of the ZnO particles from the low magnification (Figure 1a) as well as high magnification (Figure 1a,b) FESEM images. However, few ZnO structures with ellipsoidal and non-spherical shapes can also be seen. These ZnO nanoballs further form some agglomerated structures. The surface of the ZnO nanoballs is highly rough as confirmed from a close look at the high magnification FESEM image as shown in Figure 1c. The average diameter of the ZnO nanoballs is 80 ± 10 nm. The roughness of the ZnO nanoballs surface provides a high density of the active sites for the adsorption of the target analyte and O_2_ from the air. In Figure 1d the energy dispersive spectroscopy (EDS) spectrum for the hydrothermally synthesized ZnO nanoballs is shown. The presence of peaks only for Zinc and oxygen atoms confirms the formation of the ZnO along with a high degree of purity for the synthesized ZnO nanoballs.

**Figure 2:**
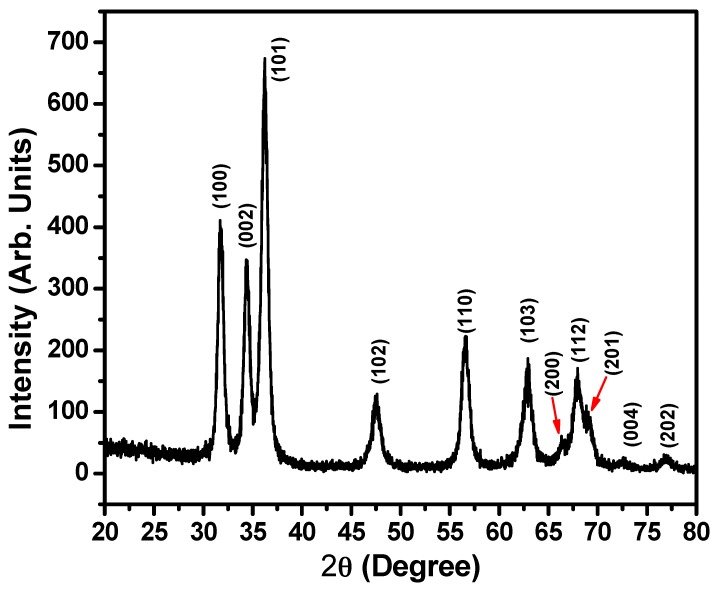
Typical XRD patterns for hydrothermally synthesized ZnO nanoballs.

The crystallinity, crystalline size and microstructural phases for the ZnO nanoballs can be evaluated from the X-ray diffraction (XRD) spectrum as shown in Figure 2. Well-defined diffractions peaks corresponding to the diffraction planes (100), (002), (101), (102), (110), (103), (200), (112), (201), (004) and (202) at diffraction angles 31.78°, 34.43°, 36.23°, 47.63°, 56.61°, 62.91°, 66.40°, 67.95°, 69.14°, 72.59° and 76.71°, respectively, indicate the Wurtzite hexagonal phase for ZnO nanoballs. The results are supported by the JCPDS data card Nos. 36–1451 and reported literature [37,38,39,40,41,42]. No additional peak in the XRD spectrum related to any impurity, further confirms the results of EDS studies (Figure 1d).

Debye–Scherrer formula (Equation (1)) was used for calculating the crystallite size (d) of the ZnO nanoballs [43].

(1) $$d=\frac{0.89\lambda }{\beta \cdot \mathrm{Cos}\theta }$$

where λ = the wavelength of X-rays used (1.54 $\overset{o}{A}$), θ is the Bragg diffraction angle and β is the peak width at half maximum (FWHM). The FWHM values for the three most intense diffraction peaks corresponding to diffraction planes (100), (002) and (101) were taken into account. The corresponding results are given in Table 1. The average crystallite size of ZnO nanoballs was found to be 10.47 nm.

**Figure 3:**
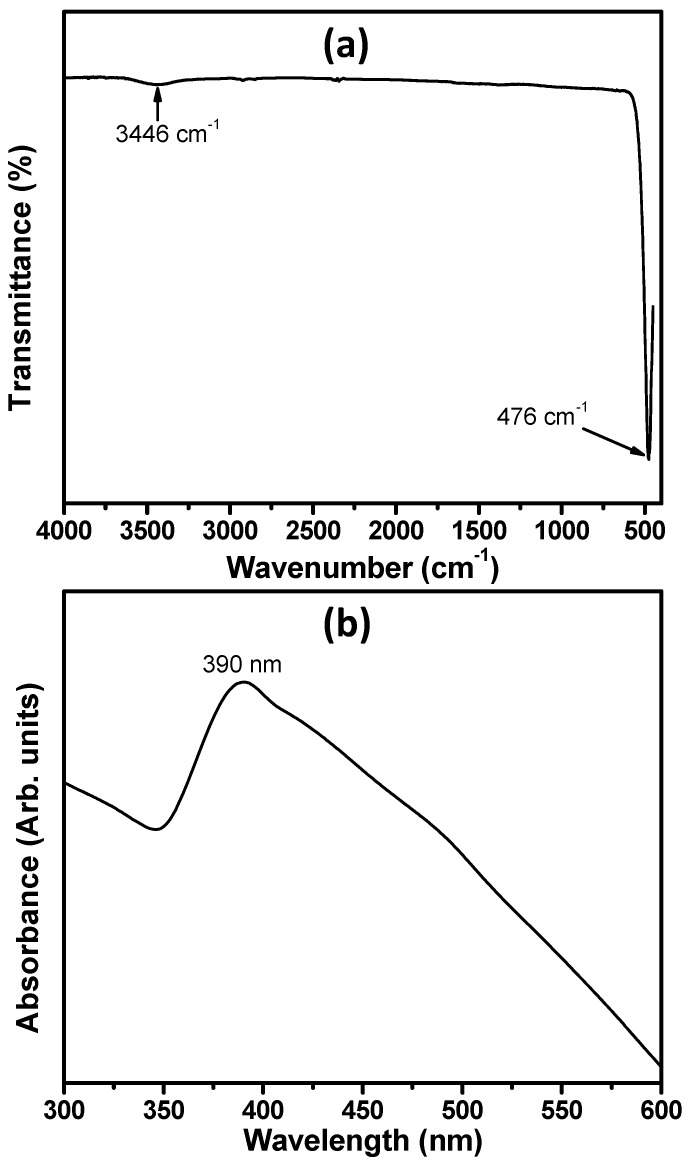
(**a**) FTIR; and (**b**) UV-Vis. spectra for hydrothermally synthesized ZnO nanoballs.

Figure 3a represents the typical Fourier transform infrared (FTIR) spectrum of hydrothermally synthesized ZnO nanoballs. A sharp and well-defined peak at 476 cm^−1^ is the characteristic peak for metal-oxygen (M–O) bond and confirms the formation of the Zn–O bond. Another broad band at 3446 cm^−1^ is due to the O–H stretching vibrational modes of the water molecules physiosorbed on the surface of the ZnO nanoballs [16,44,45,46].

In Figure 3b, the UV-Vis. spectrum plotted in the range of 200–550 nm is shown. A single and sharp absorption peak at 390 nm is observed. The band gap energy (*E_g_*) of 3.19 eV was calculated with the help of well-known Planck’s quantum equation (Equation (2)) [47].

(2) $$E_{g}=\frac{hc}{\lambda_{max}}=\frac{6.625\times 10^{-34}\;Js\times 3\times 10^{8}\;ms^{-1}}{390\times 10^{-9}\;m\times 1.6\times 10^{-19}}=3.19\;eV$$

**Figure 4:**
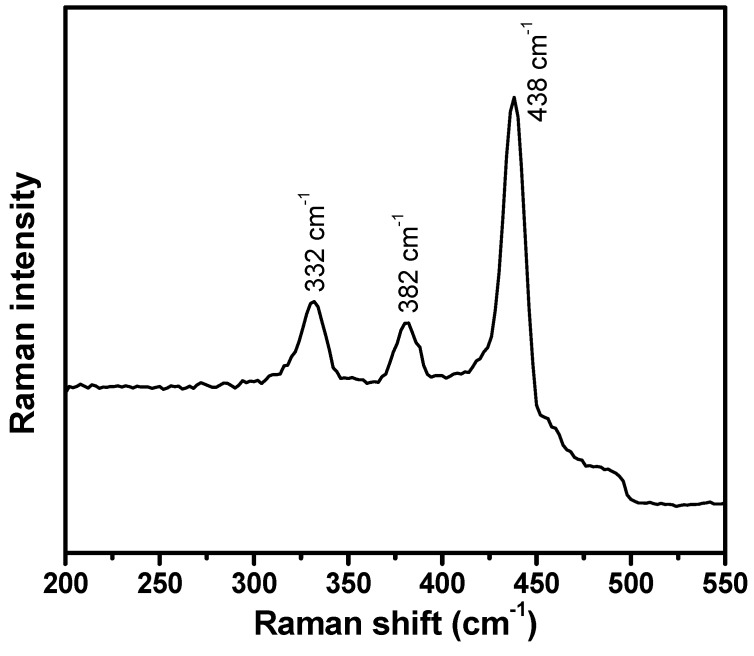
Raman spectrum for hydrothermally synthesized ZnO nanoballs.

In order to evaluate the molecular vibrational, polarization and scattering information for the ZnO nanoballs, Raman-scattering analysis was performed at room temperature. Figure 4 represents the Raman scattering spectrum of the hydrothermally synthesized ZnO nanoballs.

Three distinct phonon peaks at 332, 382 and 438 cm^−1^ are the typical characteristic peaks of the ZnO wurtzite hexagonal phase and correspond to E_2H_–E_2L_ multiphonon process, A_1_(TO) and $E_{2}^{\mathrm{High}}$ modes, respectively [48]. Stronger $E_{2}^{\mathrm{High}}$ indicates excellent crystal qualities and very low oxygen vacancies on the surface of the ZnO nanoballs [49].

### 2.2. Characterization of Acetone Sensor Fabricated Based on ZnO Nanoballs

**Figure 5:**
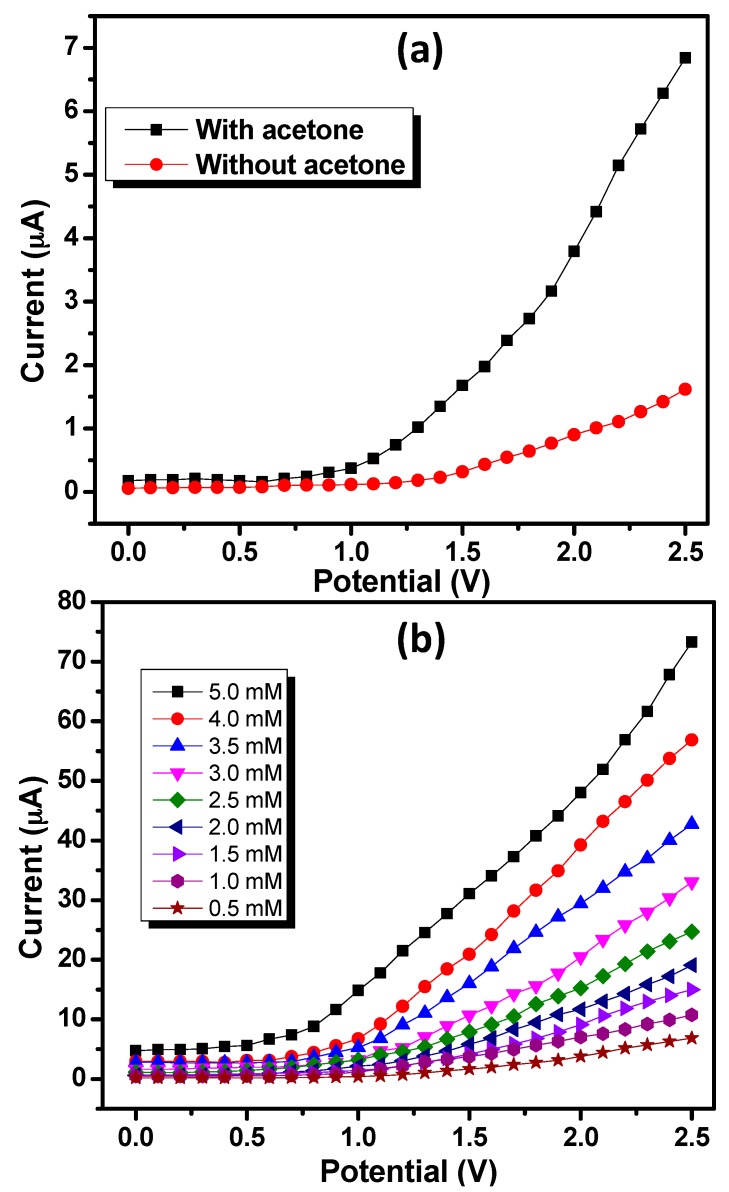
(**a**) *I*–*V* responses measured for 0.5 mM acetone in 0.1 M PBS solution and blank PBS solution using ZnO nanoballs modified AgE; and (**b**) *I*–*V* response variations for 0.5 mM–5.0 mM concentrations of acetone in 0.1 M PBS solution.

The potential electro-catalytic sensing applications of ZnO nanoballs coated onto the surface of the AgE are demonstrated in this section. Initial experimentations involves the comparison of *I*–*V* responses of the ZnO nanoballs modified AgE for 0.5 mM acetone solution prepared in the 0.1 M PBS having pH 7.4 and blank PBS within the potential range of 0.0–2.5 V. As the applied potential increases, the current response increases remarkably for the PBS containing acetone as compared to blank PBS (Figure 5a). At an applied potential of 2.5 V, the maximum current responses of 6.84221 and 1.6182 μA were observed for 0.5 mM acetone solutions and blank PBS, respectively. This substantial response of the ZnO nanoballs modified AgE towards the sensing of acetone confirms the involvement of the ZnO nanostructures in the efficient electrocatalytic activities and fast electron exchange capabilities. Figure 5b represents the effect of the acetone concentration on the current responses of the ZnO nanoballs modified AgE. Different solutions of acetone with concentration range of 0.5 mM–5.0 mM were prepared in 0.1 M PBS and were subjected to electrochemical analysis using ZnO nanoballs modified AgE as working electrode and a Pt wire as a counter electrode within the potential range of 0.0–2.5 V. It can be seen that the increase in the concentration of the acetone resulted in a marked increase in the current responses.

At an applied potential of 2.5 V, the current responses of 6.84221, 10.7272, 14.9831, 19.0696, 24.7101, 33.013, 42.7444, 56.8836 and 73.3094 µA were recorded for 0.5, 1.0, 1.5, 2.0, 2.5, 3.0, 3.5, 4.0 and 5.0 mM acetone solutions, respectively. Increased current responses with a concentration of the acetone can be attributed to the generation of a large number of ions and increased ionic strength of the analyte solutions [37].

**Figure 6:**
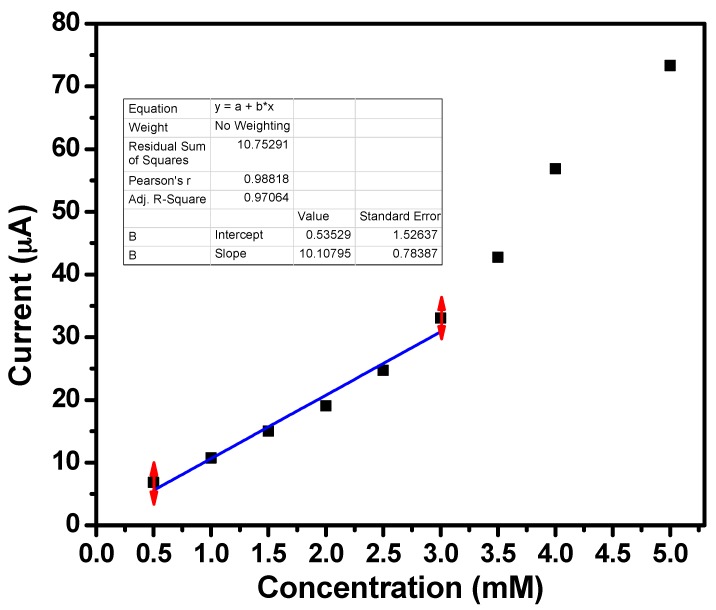
Calibration plot for ZnO nanoballs modified AgE towards acetone.

Current vs. concentration calibration graph was plotted to determine the sensing parameters such as sensitivity, LOD and LDR (Figure 6). The sensitivity value of ∼472.33 μA·mM^−1^·cm^−2^ and LDR of 0.5 mM–3.0 mM with a correlation coefficient (*R*^2^) of 0.97064 were obtained from the calibration graph. Experimental LOD for ZnO nanoballs modified AgE was found to be 0.5 mM. As fabricated acetone sensors based on hydrothermally synthesized ZnO nanoballs exhibit better sensitivity compared to different sensors reported in the literature (Table 2).

### 2.3. Proposed Sensing Mechanism

It has been postulated in many studies that the adsorption of the molecular oxygen (O_2_) from the PBS as well as from the surrounding environment onto the highly rough surface of the ZnO nanomaterials, is the key concern of the sensing applications. Surface reactions result in the formation of oxygenated anionic species such as superoxides ($O_{2}^{-}$), peroxides ($O_{2}^{2-}$), hydroxides (HO^−^) and oxides ($O^{2-}$) [57]. The reduction is aided through the conduction band electrons of the ZnO nanomaterials coated onto the surface of AgE (Equations (3)–(6)).

(3) $$O_{2\;\left(g\right)}\to O_{2\;\left(\mathrm{chemisorbed}\right)}$$

(4) $$O_{2\;\left(\mathrm{chemisorbed}\right)}+{\;e}^{-}\to {\;O}_{2\;\left(\mathrm{Chemisorbed}\right)}^{-}$$

(5) $$O_{2\;\left(\mathrm{chemisorbed}\right)}+\;2e^{-}\to {\;O}_{2\;\left(\mathrm{Chemisorbed}\right)}^{2-}$$

(6) $$O_{2\;\left(\mathrm{chemisorbed}\right)}+\;4e^{-}\to \;2O_{\;\left(\mathrm{Chemisorbed}\right)}^{2-}$$

These chemisorbed oxygenated anionic species deplete the surface electron states of the ZnO and increase the resistance of the n-type semiconductor material due to the formation of an electron depletion layer at the ZnO nanoballs surfaces [58,59,60,61]. The increase in the current response is due to the release of the trapped electrons back into the conduction band during the catalytic oxidation the adsorbed acetone molecules into CO_2_ and H_2_O (Equations (7)–(10)) [18,44,62,63,64].

(7) $${\mathrm{CH}}_{3}{\mathrm{COCH}}_{3}+4O^{-}{\;}_{\left(\mathrm{chemisorbed}\right)}\to {\mathrm{CH}}_{3}{\mathrm{COOH}}_{}+{\mathrm{CO}}_{2}+H_{2}O+4e^{-}$$

(8) $${\mathrm{CH}}_{3}\mathrm{COOH}+4O^{-}{\;}_{\left(\mathrm{chemisorbed}\right)}\to 2{\mathrm{CO}}_{2}+2H_{2}O+4e^{-}$$

(9) $${\mathrm{CH}}_{3}{\mathrm{COCH}}_{3}+4O_{2}^{-}{\;}_{\left(\mathrm{chemisorbed}\right)}\to 3{\mathrm{CO}}_{2}+3H_{2}O+4e^{-}$$

(10) $${\mathrm{CH}}_{3}{\mathrm{COCH}}_{3}+8O^{2-}{\;}_{\left(\mathrm{chemisorbed}\right)}\to 3{\mathrm{CO}}_{2}+3H_{2}O+16e^{-}$$

**Figure 7:**
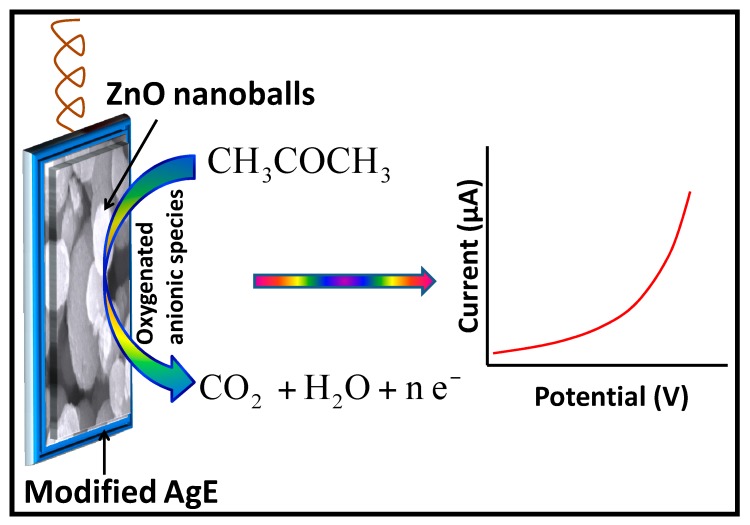
Proposed sensing mechanism for ZnO nanoballs modified AgE towards acetone in PBS.

On the basis of above discussion, the proposed sensing mechanism for ZnO nanoballs modified AgE against acetone is represented in Figure 7.

Thus, ZnO nanoballs pose excellent electron mediator activities for the detection and sensing of very low level of acetone in PBS at room temperature.

## 3. Materials and Methods

### 3.1. Hydrothermal Synthesis of ZnO Nanoballs

For the synthesis of the ZnO nanoballs, all chemicals were purchased from Sigma–Aldrich (St. Louis, MO, USA) and were used as received without any further refinement. All solutions were prepared in DI water. A facile hydrothermal method was adopted for the synthesis of ZnO nanoballs in which 50 mL of 0.02 M Zinc nitrate hexahydrate [Zn(NO_3_)_2_·6H_2_O] was continuously stirred for 30 min along with aqueous NaOH solution, added dropwise in order to maintain a pH of 10. Thereafter the resulting solution was transferred to a Teflon-lined stainless steel autoclave which was heated to 150 °C. After heating the autoclave for the desired growth time oh for 5 h, it was slowly cooled to room temperature. The white product formed was filtered and washed with DI water and ethanol to remove any un-reacted reactants. Finally, the powder was dried at 70 ± 2 °C for 2 h in a hot air oven and characterized for its morphological, optical, structural, compositional and electrochemical sensing applications using different analytical techniques.

### 3.2. Fabrication of Acetone Sensor Based on ZnO Nanoballs

The silver electrode of active surface area of 0.0214 cm^2^ was pre-cleaned using 0.05 μm alumina slurry followed by thorough washings with distilled water and ethanol and finally dried for 1 h in hot air oven at 70 °C. A homogeneous thin paste of ZnO nanoballs was prepared in butyl carbital acetate (BCA) conducting solvent and was coated in the form of a thin layer over the surface of the Ag electrode. The coated Ag electrode was dried at 70 °C in an air oven for 4 h. An electrochemical cell was then set up in which ZnO nanoballs modified AgE served as the working electrode and a Pt wire as the counter electrode. The current–voltage (*I*–*V*) measurements for solutions of acetone with different concentrations were measured at room temperature in the presence of 0.1 M phosphate buffer solution (PBS) with pH of 7.4 with the help of Keithley 6517A-USA electrometer (Tektronix, OR, USA) with computer interfacing. The acetone sensitivity was determined by generating a calibration curve of current vs. concentration. The sensitivity of the ZnO nanoballs modified AgE was determined from the ratio of the slope of the calibration graph plotted between current and concentration to the active surface area of modified AgE.

### 3.3. Characterization of ZnO Nanoballs

Field emission scanning electron microscopy (FESEM; JEOL-JSM-7600F, JEOL, Tokyo, Japan) integrated with EDS was examined in order to study the morphological, compositional, and structural properties of the hydrothermally synthesized ZnO nanoballs. X-ray diffraction (XRD; JDX-8030W, JEOL, Tokyo, Japan) studies were conducted between the diffraction angles (2θ) range of 20°–80° using Cu-Kα source radiation with a wavelength of 1.54 Å in order to explore the crystallinity, crystalline size, and microstructural phases. UV-visible spectrophotometer (Perkin Elmer-UV- -Vis. Lambda 950, PerkinElmer, MA, USA) analysis of the aqueous solution of ZnO nanoballs, sonicated for 15 min was carried out between the scan range of 300–600 nm to evaluate the band gap energy and optical properties. Fourier transform infrared spectroscopic (FTIR; Perkin Elmer-FTIR Spectrum-100, PerkinElmer, MA, USA) analysis was conducted in order to analyze the composition of the as-synthesized ZnO nanoballs. Raman-scattering spectroscopic (Perkin Elmer-Raman Station 400 series, PerkinElmer, MA, USA) technique was utilized for the examination of scattering properties of the ZnO nanoballs in the scan range of 200–550 cm^−1^.

## 4. Conclusions

A simple, low cost, template-free hydrothermal method was adopted for the synthesis of ZnO nanoballs with highly rough surfaces. Morphological, structural, optical, crystal phases, vibrational and scattering properties of the ZnO nanoballs were evaluated through different analytic techniques such as FESEM, EDS, UV-Vis, FTIR and Raman scattering spectroscopy. ZnO nanoballs were further utilized for the fabrication of highly sensitive acetone electrochemical sensors through *I*–*V* techniques. All the observations were recorded at room temperature and in the presence of the 0.1 M PBS with pH of 7.4. High sensitivity of ~472.33 μA·mM^−1^·cm^−2^ and LDR of 0.5 mM–3.0 mM were obtained. Hence, ZnO nanoballs based electrochemical sensors may introduce a relatively new avenue for the fabrication of efficient sensor for hazardous and carcinogenic chemicals and in environmental and healthcare monitoring.

## Figures and Tables

**Table 1:** The crystallite size of the hydrothermally synthesized ZnO nanoballs.

S.N	(hkl)	2θ (°)	FWHM (β)	Crystallite Size (nm)
1	(100)	31.78	0.71936	11.36
2	(002)	38.43	0.80871	10.18
3	(101)	36.23	0.83756	9.88

**Table 2:** Summary of the acetone sensing performances of different sensor materials.

Sensor	Method	Sensitivity	LDR	LOD	R^2^	Ref.
ZnO-doped Co_3_O_4_ Nanorods/AgE	*I*–*V*	3.58 μA·mM^−1^·cm^−2^	66.8 μM–0.133 mM	14.7 ± 0.2 μM	0.9684	[50]
ZnO NPs/GCE	*I*–*V*	0.14065 μA·mM^−1^·cm^−2^	0.13 mM–0.13 M	0.068 ± 0.01 mM	-	[51]
Gd-ZnO-Nanopencils/AgE	*I*–*V*	208 ± 62 μA·mM^−1^·cm^−2^	750 μM–100 mM	0.7 mM	0.885	[52]
ZnO/SnO_2_/Yb_2_O_3_/GCE	*I*–*V*	17.09 μA·mM^−1^·cm^−2^	0.34 nM–3.4 mM	0.05 ± 0.002 nM	0.9394	[53]
Lead foil electrode	Amperometric	2.07 μA·cm^−2^·ppm^−1^	50–250 ppm	50 ppm	0.998	[54]
Electro-deposited Pb electrode	Amperometric	4.16 μA·cm^−2^·ppm^−1^	100–400 ppm	-	0.99	[55]
Ag_2_O microflower/GCE	*I*–*V*	1.699 μA·mM^−1^·cm^−2^	0.13 μM–0.67 M	0.11 μM	0.9462	[56]
***ZnO nanoballs/AgE***	***I***–***V***	***472.33*** ***μA***·***mM^−1^***·***cm^−2^***	***0.5 mM–3.0 mM***	***0.5 mM***	***0.9706***	***This work***
